# The roles of salivary secretory IgA on the development of oral candidiasis

**DOI:** 10.3389/froh.2026.1760095

**Published:** 2026-03-04

**Authors:** Jingzhi Zhou, Jiannan Wang, Jiawei Shen, Yifan Lin, Lunwei Kang, Yunting Wang, Yujie Zhou, Ga Liao, Biao Ren

**Affiliations:** 1 State Key Laboratory of Oral Diseases, National Center for Stomatology, National Clinical Research Center for Oral Diseases, West China School of Stomatology, Sichuan University, Chengdu, Sichuan, China; 2 West China Lecheng Hospital, Sichuan University, Boao, Hainan, China; 3 Guangdong Provincial Key Laboratory of Stomatology, Guanghua School of Stomatology, Sun Yat-Sen University, Guangzhou, China; 4 Tianfu Jiangxi Laboratory, Chengdu, Sichuan, China

**Keywords:** *Candida albicans*, oral candidiasis, oral mucosal immunity, salivary sIgA, virulence

## Abstract

Oral candidiasis, an opportunistic fungal infection mainly caused by *Candida albicans*, is highly prevalent in immunocompromised individuals. Saliva acts as the oral cavity's first line of defense, with secretory immunoglobulin A (sIgA) as its key specific immune component. In this review, we systematically clarify sIgA's multifaceted roles in oral immunity and its significance in the pathogenesis, progression, and management of oral candidiasis. We detail sIgA's biological characteristics (synthesis, secretion) and core mechanisms: immune exclusion (inhibiting fungal adhesion/invasion), virulence factor neutralization, biofilm interference, and immune regulation. We also explore sIgA-*C. albicans* interactions, including antigen recognition, hyphal transition inhibition, and fungal evasion strategies (protease degradation, antigenic variation). Clinical evidence shows that compromised salivary sIgA levels/function—due to systemic diseases (e.g., HIV/AIDS, Sjögren's syndrome), aging, radiotherapy, or immunosuppression — correlates with increased susceptibility and severity of oral candidiasis, with functional quality being equally crucial as quantity. Given conventional antifungal limitations, we discuss sIgA-based interventions (recombinant sIgA passive immunization, mucosal vaccines, probiotic adjuvants). In conclusion, salivary sIgA is critical to maintaining oral mucosal homeostasis against *C. albicans*, and enhancing its function offers promising avenues for preventing and treating oral candidiasis.

## 1 Introduction

Oral candidiasis is one of the most common opportunistic fungal infections of the oral mucosa, primarily caused by *Candida* species overgrowth, with *C. albicans* as the main pathogen (1, 2). It disproportionately affects individuals with impaired oral homeostasis or immunity, such as those with HIV infection, head and neck radiotherapy, xerostomia, denture wearers, or broad-spectrum antibiotic users, as these factors disrupt local defense and microbial balance (1, 3). The oral mucosal immune system, comprising innate and adaptive components, plays a critical role in maintaining oral health and preventing candidiasis. Saliva, a key defense component, contains various antimicrobial agents, among which sIgA is the dominant salivary immunoglobulin (4, 5). In humans, IgA is the dominant antibody class at mucosal surfaces, and sIgA is the principal immunoglobulin on these surfaces (4, 6). As the most crucial specific defense factor of saliva, sIgA plays a universal role in mucosal immunity and is essential in local (oral mucosal) immunity (4, 5). Several studies have confirmed the local production and functional significance of salivary sIgA (7), further supporting its key role in oral mucosal immune defense. Synthesized mainly by salivary gland plasma cells, sIgA performs multiple key functions in oral mucosal defense. In the oral cavity, sIgA exerts multifaceted antifungal effects, it provides immune exclusion by agglutinating pathogens and blocking their adhesion to epithelial surfaces (4, 8). SIgA also neutralizes microbial toxins, viruses, and enzymes (8, 9). Furthermore, sIgA disrupts biofilm formation and collaborates with other salivary components, such as mucins, to enhance microbial clearance and maintain oral mucosal homeostasis (10, 11). Salivary sIgA can also cooperate with multiple immune cells (such as neutrophils, phagocytes, etc.) to regulate immune effects (9, 12). Its vital role is underscored by clinical observations where factors like xerostomia (dry mouth) reduce salivary flow, adversely affecting sIgA concentration and function, and increasing susceptibility to infections such as oral candidiasis (13). Despite sIgA's crucial role in mucosal and oral immunity, its specific functions in oral candidiasis, interactions with *C. albicans*, and the implications of quantitative and qualitative variations on disease susceptibility and progression require comprehensive examination. Moreover, conventional antifungal therapies face limitations such as rising drug resistance (especially in biofilm-associated infections) and potential toxicity, creating an urgent need for alternative strategies (14, 15). This review focuses on the interplay between salivary sIgA and oral candidiasis, exploring sIgA's biological functions, factors modulating its levels, and prospects for sIgA-based therapeutic approaches.

## 2 Mucosal immunity and oral candidiasis

Oral candidiasis arises from imbalances in oral homeostasis, impaired local defense, immune disturbances, and oral microbiome dysbiosis, facilitating pathogenic fungal dissemination and biofilm formation (3, 13). Susceptible individuals include those with poor oral hygiene, xerostomia, HIV infection, head/neck cancer post-radiation, immunosuppression, salivary dysfunction, denture use, or corticosteroid/antibiotic therapy (1, 16, 17). Additional risks encompass high-carbohydrate diets, age extremes, smoking, diabetes, Cushing's syndrome, and malignancies (1). The disease exhibits diverse manifestations, categorized as primary (e.g., pseudomembranous, atrophic, hyperplastic, chronic atrophic forms) or secondary candidiasis (18). Presentations range from acute to subacute or chronic (19–21). Symptoms like oral pain, burning, taste alterations, dysphagia, malnutrition, and prolonged hospitalization (1, 22, 23). It is a common opportunistic infection caused by *Candida* overgrowth (predominantly *C. albicans*), representing the most frequent human fungal infection, especially in early and late life (17, 19, 20).

The oral mucosa is a key physiological and immunological barrier, relying on innate and adaptive immunity (13, 24). Epithelial cells recognize *C. albicans* via pattern recognition receptors, triggering proinflammatory cytokines that recruit neutrophils and macrophages, and initiate T cell-mediated adaptive immunity, particularly the crucial Th17 response (3, 25). In health, Tregs provide immunomodulation, while Th17 cells, often with tongue-resident γδ T cells, maintain baseline mucosal defense by secreting IL-17 to regulate commensals like *Candida*. During infection, this defense intensifies: the Th17/IL-17 axis promotes granulopoiesis and neutrophil recruitment for pathogen clearance, while cytotoxic T lymphocytes (CTLs) are recruited to lyse infected cells. Tresp cells induced by Tregs can also enhance clearance via IL-17 (26). Th17 cells are indispensable for antifungal immunity; genetic defects in this pathway increase susceptibility to chronic mucosal fungal infections (14). Salivary sIgA, the body's largest humoral immune system, is the major antibody at mucosal surfaces and serves as a critical first line of defense against pathogens (27).

Saliva includes various antimicrobial agents and plays important roles in resistance to infection by *C. albicans* in the oral cavity, with salivary protein flow influencing initial colonization (28). It contains multiple kinds of antimicrobial proteins and peptides, including non-specific immune components such as lysozyme, lactoferrin, lactoperoxidase, histatins, defensins, and calprotectin, and specific immune components, particularly sIgA (29). Saliva not only dilutes and clears candidal species from the oral cavity but also discourages proliferation through those immune components and anti-*Candida* antibodies. Lactoferrin inhibits *Candida* cell growth by sequestering iron necessary for proliferation (30). Histatins are the major antifungal proteins in human saliva (31). SIgA is the main type of antibody present in saliva. Saliva and its constituents maintain oral eco-balance and health via debridement and lavage, aggregation and suppressing adherence of organisms, and direct antibacterial and antifungal activities (32).

## 3 Roles of sIgA in mucosal immunity

### 3.1 SIgA synthesis and secretion

SIgA is a fundamental immunoglobulin in mucosal immunity. Its core structure is shared with other antibodies, consisting of two identical heavy chains and two identical light chains. Each heavy/light chain pair forms a Fab region responsible for antigen binding, while a flexible hinge region connects these to an Fc region that mediates effector functions (33). At mucosal surfaces like the oral cavity, salivary sIgA predominantly exists in a polymeric form, which corresponds to mucosal site-specific dimeric IgA (dIgA) that is composed of two monomeric units covalently linked by a joining (J) chain (34).

The synthesis and secretion of salivary sIgA are highly localized. It is primarily produced by mucosal plasma cells and antibody-secreting cells (ASCs) derived from B cells residing within the salivary glands and the lamina propria of the oral mucosa, operating independently of the gut-associated lymphoid tissue (GALT) (5, 7, 11, 13, 35). Following synthesis, dIgA is transported across the epithelium via transcytosis. This critical process is mediated by the polymeric immunoglobulin receptor (pIgR), synthesized by the epithelial cells themselves (11, 36). Upon release, the extracellular portion of the pIgR, known as the secretory component (SC), remains bound to the IgA complex, forming the complete and stable sIgA molecule (4, 6, 37). The bound SC is essential for sIgA's remarkable resilience. It masks vulnerable sites on the IgA molecule, providing robust protection against proteolytic degradation in the enzymatically hostile environment of the oral cavity, thereby preserving its functionality (4, 6, 27, 38). Functionally, sIgA is the major immunoglobulin and primary specific defense factor in saliva. It primarily targets oral pathogens (e.g., *Streptococcus, Candida*), dietary antigens, and toxins, playing a pivotal role in maintaining oral microbial homeostasis through immune exclusion and neutralization (4, 39).

### 3.2 Functions of sIgA

Salivary sIgA is the principal antibody defending oral mucosal surfaces through multiple coordinated mechanisms. Its functional integrity relies on its unique structure, particularly the J chain and SC. The J chain is essential for forming dIgA and binding to the pIgR for transcytosis. SC, derived from cleaved pIgR, covalently binds to IgA, protecting it from proteolysis and enhancing its interaction with mucosal components like mucins, thereby optimizing immune exclusion (4, 11, 27, 36, 38).

A primary defensive function is immune exclusion. SIgA cross-links environmental microorganisms, preventing their adhesion and invasion. Specifically, it can directly bind to mannans on the cell wall of *C. albicans*. By occupying adhesins binding sites on the fungal surface, it prevents adhesins from interacting with epithelial cells, thereby inhibiting the mucosal adhesion of *C. albicans* (8, 36, 40, 41). The direct binding of sIgA to *C. albicans* inhibits its adhesion and invasion, reduces the stimulation of epithelial cells, and then down-regulates the secretion of pro-inflammatory mediators such as CXCL8/IL-8, IL-1α and IL-1β by epithelial cells, maintains mucosal homeostasis, and avoids tissue damage caused by excessive inflammation (24, 42). SIgA has been verified *in vitro* experiments to neutralize virulence factors like viruses (such as HIV) and bacterial toxins (such as lipopolysaccharide) (8, 9). During pIgR-mediated transport, it can even inactivate viruses intracellularly to prevent the damage in cells (27). Within the oral cavity, salivary sIgA interferes with biofilm formation by inhibiting microbial colonization and disrupting the initial steps of biofilm maturation on surfaces like dental plaque, helping maintain microbial balance (8, 10, 11).

Salivary sIgA does not act in isolation but synergizes with other salivary components. It binds to mucins to form a stable mucus barrier that traps pathogens and facilitates clearance via saliva flow. This interaction prolongs salivary sIgA's residence time, enhancing its functions (4, 11). Collaboration with antimicrobial peptides could further inhibits pathogen growth. Furthermore, sIgA actively interacts with immune cells to modulate immunity. It can influence dendritic cell maturation and guide T-cell differentiation towards regulatory subtypes, supporting mucosal tolerance (43). SIgA-antigen complexes can be presented by dendritic cells to activate CD8^+^ T cells, linking humoral and cellular immunity (44). Engagement of FcαRI (Fcα receptor I) on neutrophils and macrophages by sIgA triggers antimicrobial responses: it promotes antibody-dependent cellular phagocytosis (ADCP) of pathogens like *C. albicans* and can induce antibody-dependent cellular cytotoxicity (ADCC) (9, 12). Human NK cells express a novel IgA receptor, which could binds to sIgA and specifically regulates the killing activity of NK cells against target cells (45). These interactions are crucial for clearing pathogens from mucosal surfaces.

## 4 Relationships between sIgA and oral candidiasis

### 4.1 SIgA deficiency and oral candidiasis

Clinical studies demonstrate a clear inverse relationship between salivary sIgA levels and oral candidiasis severity. Patients exhibit significantly lower salivary sIgA concentrations and excretion rates compared to healthy individuals, a deficit linked to impaired production by B cells and plasma cells (40). *C. albicans* itself can directly suppress human oral mucosal epithelial cells’ secretion of IgA (2). Consequently, IgA-deficient individuals face a markedly higher frequency of candidal infections (36, 46).

Multiple systemic diseases and conditions profoundly influence salivary sIgA levels and function, thereby impacting candidiasis risk. In HIV/AIDS, salivary concentrations of total IgA and subclasses (especially IgA2) are significantly reduced, with declines worsening as the disease progresses; diminished salivary flow further compromises mucosal defense (47). Sjögren's syndrome impairs salivary gland function, leading to hyposalivation, decreased salivary sIgA, and increased *Candida* carriage (11, 46). Similarly, diabetes mellitus promotes *Candida* colonization through elevated salivary glucose, while patients have the phenomenon of decreased saliva secretion (5). Other conditions like autoimmune diseases and malignancies also indirectly weaken sIgA-related immunity (18).

Medical interventions are major modifying factors. Immunosuppressants (e.g., corticosteroids, chemotherapy) and broad-spectrum antibiotics alter salivary proteins including sIgA and disrupt microbiome balance, predisposing to infection (13, 18). Head and neck radiotherapy (HNRT) damages salivary glands, causing hyposalivation, xerostomia, reduced salivary sIgA levels, and significantly increased *Candida* colonization and candidiasis incidence (3, 15, 48). Cancer patients undergoing such therapies exhibit markedly lower salivary sIgA levels and salivary flow, which enhances the salivary microbial load (49, 50). *C. albicans* is the predominant pathogen in these settings.

**Figure 1:**
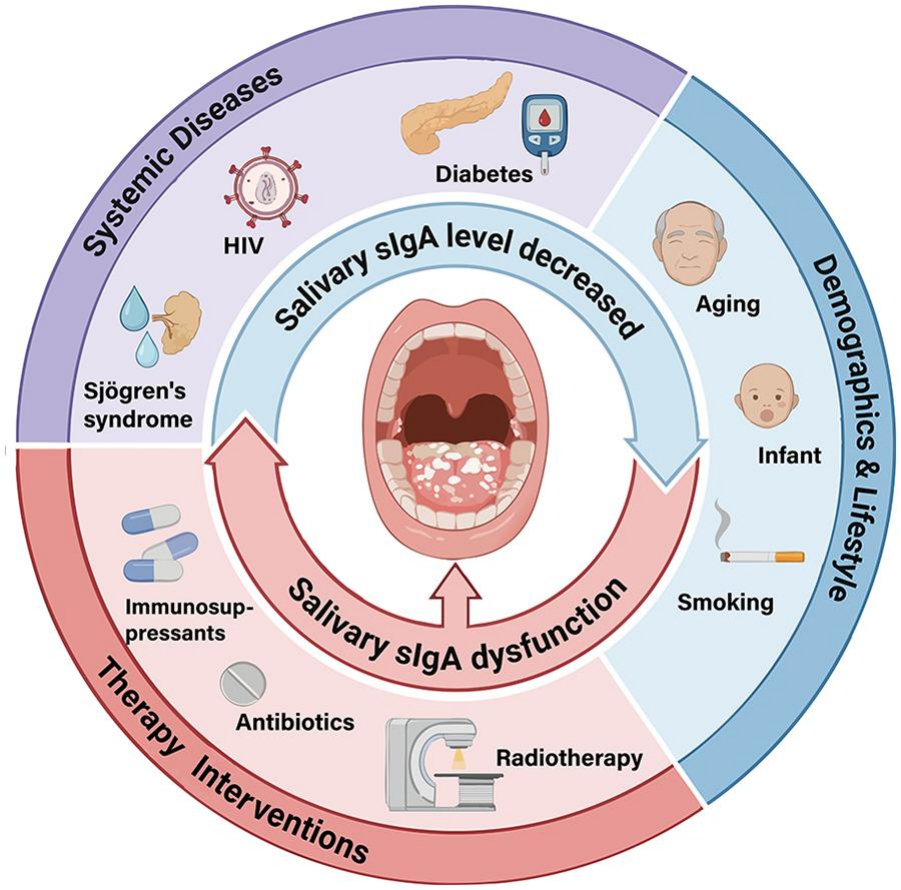
Factors predisposing to oral candidiasis by affecting salivary sIgA levels or function. Created in BioRender. Salivary total IgA subclass were significantly decreased in HIV/AIDS patients. Sjögren's syndrome patients and head and neck radiotherapy recipients both presented hyposalivation and reduced salivary sIgA levels, with salivary gland injury as the major cause in the latter group. Salivary sIgA levels showed an independent decrease in smokers. Elderly individuals (denture wearers), infants and diabetic patients exhibited reduced salivary sIgA levels, which were associated with hyposalivation, immature immune system and elevated salivary glucose respectively. Increased salivary sIgA levels in special populations (HIV-Positive children, patients with uncontrolled diabetes, and patients with denture stomatitis) are consistent with a higher risk of *Candida* infection, suggesting that sIgA function may be impaired in these populations.

Age can affect salivary sIgA mediated mucosal defense: salivary gland dysfunction and decreased salivary flow in the elderly weaken the protective effect of salivary sIgA, which is also associated with diseases such as denture stomatitis (a common subtype of oral candidiasis in the elderly population). Infants are also susceptible to oral candidiasis (11, 18, 51). Salivary sIgA concentrations also follow circadian rhythms. Critically, lifestyle like tobacco smoking independently reduces salivary sIgA levels, induces epithelial keratinization, and impairs neutrophil function, all favoring *Candida* colonization and establishing smoking as a key risk factor for clinical candidiasis (46, 52). These predisposing factors contributing to oral candidiasis by affecting salivary sIgA levels or function are all shown in Figure 1.

### 4.2 Interactions between *Candida albicans* and sIgA

As a commensal, *C. albicans* colonizes 30%–70% of healthy individuals (24). *C. albicans* initiates a pathogenic switch through hyphal formation and secretion of virulence factors, leading to *Candida* overgrowth and potential systemic spread in immunocompromised hosts (23, 53). While clinical studies frequently correlate reduced salivary secretory sIgA levels with the severity of oral candidiasis, conflicting findings have also been documented. For instance, elevated salivary sIgA levels are observed in certain populations (e.g., HIV-positive children, patients with uncontrolled diabetes, and individuals with denture stomatitis), who concurrently display an increased risk of oral candidiasis. This paradox may arise from sIgA functional impairment, such as decreased antibody avidity or an insufficient compensatory rise during active infection, rather than mere concentration decreased (46, 54–57). These controversies underscore that both the “quality” (functional attributes) and “quantity” (concentration) of sIgA are critical for mucosal defense, with functional parameters like avidity and specificity being as important as absolute levels (4, 11). Differences in salivary sIgA indicators and disease incidence between healthy individuals and susceptible to oral candidiasis are shown in Table 1.

**Table 1:** Summary of salivary sIgA indicators and disease incidence in healthy and oral candidiasis-susceptible populations.

Population	Indicator type	Comparison	Ref.
Health	sIgA concentration	Elevated in candidiasis	(46)
	Anti-adhesion activity	Effective inhibition of *C. albicans* adhesion	(54)
	Antigen affinity	Salivary IgA affinity (no disease): 74 ± 2	(57)
	Disease incidence	Low clinical incidence; higher in IgA-deficient individuals	(46, 54)
HIV	sIgA concentration	Decline as HIV infection progresses to AIDS	(54)
	Anti-adhesion activity		
	Antigen affinity	Higher when no disease (81 ± 1.6); lower with AIDS & disease (72 ± 3.8)	(57)
	Disease incidence	47.5% in AIDS; higher colonization	(73)
Diabetes	sIgA concentration	Higher in uncontrolled; no difference if controlled	(55, 74)
	Anti-adhesion activity		
	Antigen affinity		
	Disease incidence	Higher; yeast counts increased (30% vs 17% healthy);	(74)
Radiotherapy	sIgA concentration	Reduced due to decreased salivary secretion	(46)
	Anti-adhesion activity	Insufficient to inhibit colonization	(46)
	Antigen affinity		
	Disease incidence	Higher, colonization increased	(46)

SIgA targets a variety of *C. albicans* antigens. It preferentially binds to the hyphal morphotype, targeting hypha-enriched cell-surface adhesins such as Als1, Als3, and Hwp1, which are key mediators of host tissue adherence (53). SIgA also targets fungal lectin-like protein. Specific epitopes recognized include mannan and specific mannoproteins like phosphoglycerate kinase and fructose bisphosphate aldolase (58, 59).

A key protective mechanism of sIgA is the inhibition of the yeast-to-hyphal transition, a critical virulence step. SIgA effectively suppresses hyphal growth and adhesion. Molecularly, sIgA reduces the ergosterol content of *C. albicans* and feedback-upregulates the expression of the ergosterol biosynthesis pathway (including *ERG3*, *ERG11*, etc.), thereby inhibiting hyphal development. *In vitro* experiments, exogenous supplementation of ergosterol can reverse this phenomenon, while *in vivo* experiments have confirmed that sIgA significantly inhibits the adhesion and virulence of *C. albicans* (60). *C. albicans* can control the appropriate level of hyphal exposure through the expression of *NRG1*, thereby inducing a specifically targeted and non-destructive IgA immune response in the mucosal system. This response is able to suppress uncontrolled *C. albicans* hyperproliferation without triggering either excessive inflammation to damage the host or strong immune clearance, thus maintaining a state of “host-fungal commensal homeostasis” (23). Compared to serum IgG, sIgA exerts a more significant inhibitory effect on hyphal growth and epithelial damage (60).

SIgA also exerts regulatory effects on fungal metabolic activity and colonization: it disrupts metabolic homeostasis by interfering with ergosterol biosynthesis (60), and exerts a dose-dependent inhibitory effect on the adhesion of *C. albicans* to oral epithelial cells, thereby preventing the subsequent invasion of host cells (24, 60). Furthermore, the interaction between sIgA and *C. albicans* dampens the epithelial secretion of pro-inflammatory mediators (e.g., CXCL8/IL-8, IL-1α, IL-1β), indirectly creating an unfavorable microenvironment for colonization (24).

**Figure 2:**
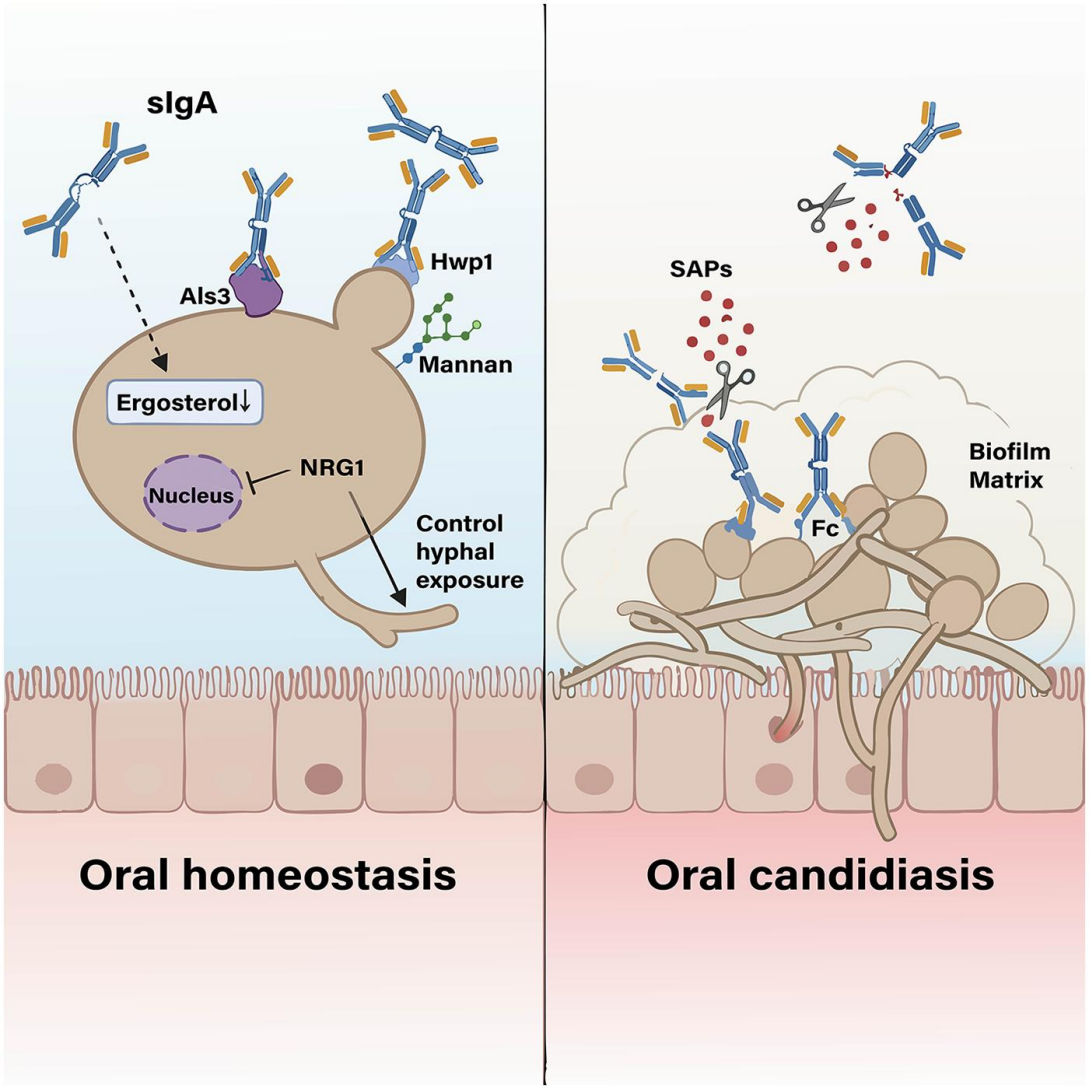
Mechanisms of interaction between sIgA and *C. albicans.* Created in BioRender. SIgA can bind to *C. albicans* cell wall components Hwp1, Als3 and mannan, inhibit fungal adhesion, reduce ergosterol content, and reduce fungal virulence. *C. albicans* regulates the expression of *NRG1* to control hyphal exposure, thereby maintaining a mild IgA-mediated immune response. SAPs secreted by *C. albicans* can degrade sIgA structures, and its biofilm matrix physically blocks sIgA contact with it.

Conversely, *C. albicans* employs multiple strategies to evade sIgA-mediated immunity. It secretes secreted aspartyl proteinases (SAPs) that degrade sIgA by cleaving peptide bonds in it, inactivating its protective function and facilitating fungal adherence and invasion (61). Antigenic variation also serves as an escape mechanism. During germ tube formation, the release of cell wall mannoproteins alters surface antigens, further lowering sIgA reactivity and aiding immune evasion (62). *C. albicans* growth in acidic saliva significantly reduces sIgA reactivity, potentially explaining the link between low salivary pH and candidiasis (63). *C. albicans* forms biofilms, whose dense extracellular matrix could physically impede sIgA penetration, preventing it from reaching and neutralizing underlying fungal cells (13). The mechanism of sIgA interaction with *C. albicans* is illustrated in Figure 2.

Salivary sIgA can bind to secretory mucins in saliva and transmembrane mucins (e.g., MUC1) on the surface of oral mucosal epithelial cells, participating in the formation of a mucus layer that covers the oral mucosa, selectively recruit specific bacteria (e.g., certain *Streptococcus* species) to anchor within it, forming a stable beneficial microbial community, occupying the ecological niche of pathogenic bacteria, and inhibiting the formation of harmful biofilms (11). Furthermore, probiotics (such as *Lactobacillus* and *Bifidobacterium*) and prebiotics can indirectly promote sIgA secretion and enhance its function by regulating the oral microbiota, holding potential roles in sIgA-based immunological interventions—probiotics can induce cytokine secretion, promote the production of immunoglobulins (including IgA, IgG, IgM) and antimicrobial substances, and induce sIgA synthesis to correct microbiota imbalance, thereby improving the defense function of epithelial cells (20, 64, 65). As a core effector molecule of oral mucosal immunity, salivary sIgA further regulates the balance of the oral microbiota through multiple mechanisms to indirectly alleviate *Candida* overgrowth: first, it exerts weak interference on the adhesion of beneficial bacteria such as *Lactobacillus* and *Bifidobacterium*, protecting these beneficial bacteria from immune clearance and promoting their mucosal colonization to enhance their competitive advantage—metabolites including lactic acid (lowering the local oral pH to 4.0–4.5), lactocidin, and hydrogen peroxide produced during the proliferation of these beneficial bacteria can further inhibit the nutrient uptake and virulence expression of *Candida* (20, 66–68); second, sIgA can regulate the structure of the oral microbiota, break the co-aggregation interaction between *Candida* and auxiliary pathogenic bacteria such as *Streptococcus mutans*, reduce the formation of synergistic pathogenic biofilms, and indirectly weaken the excessive proliferation ability and pathogenicity of *Candida* (20, 68).

## 5 Potential treatment strategies of sIgA

Conventional antifungal therapies face significant limitations, including drug resistance, toxicity, variable efficacy and drug resistance being a major and disconcerting concern (14, 49, 64). Multidrug resistance in *Candida* species is mediated by mechanisms such as efflux pumps, enzyme modifications, and biofilm formation, and it is particularly problematic in biofilm-associated infections (13, 64). Non-*albicans Candida* species (NACS) may be even more resistant to conventional antifungal drugs than *C. albicans* strains (15, 49).

These challenges drive the exploration of sIgA-based immunotherapeutic strategies. Passive immunization involves the local application of preparations containing high-titer anti-*Candida* sIgA, such as “immune milk” or recombinant sIgA produced via platforms like plant pharming (41). The expression systems for recombinant sIgA mainly include mammalian cell systems centered on Chinese hamster ovary (CHO) cells and plant-based systems using tobacco, *Arabidopsis thaliana*, duckweed (Lemna) and other carriers. The former is technologically mature, with post-translational modifications close to those of natural human sIgA and low immunogenicity, but its yield is far from meeting the clinical application benchmark of therapeutic IgG, and large-scale production relies on complex cell culture and purification processes with high costs. The latter has the advantages of low cost, scalable cultivation, no mammalian virus contamination, and simplified purification for oral application, with some systems (such as the Lemna-based LEX system) showing prominent yields (69). And a study have shown that the production of recombinant sIgA can be greatly increased by increasing the volume of ER by regulating plant phospholipid synthesis (CCT gene editing) and co-expression with molecular chaperones (70). But it faces problems including incomplete assembly, heterogeneous expression levels, and differences in glycosylation modifications compared with mammals. In terms of clinical trials, only CaroRx, a tobacco-derived anti-dental caries sIgA product, has completed human Phase I/II trials and been approved as a medical device by the European Union (69). Current recombinant sIgA research and development targets bacteria or viruses, and there are no recombinant sIgA products targeting fungi that have passed clinical trials. Given the unmet clinical needs for mucosal infections caused by fungi, this area holds promising research directions and application prospects.

Active immunization aims to develop mucosal vaccines (e.g., NDV-3A targeting the adhesin Als3) that induce specific salivary sIgA production, offering protection in preclinical models (71). Probiotic interventions (e.g., *Lactobacillus, Bifidobacterium*) and prebiotics also show promise. They can enhance epithelial defense, modulate the oral flora, serving as an adjuvant treatment for oral candidiasis and increase anti-*Candida* and total salivary sIgA levels, thereby reducing *Candida* prevalence, especially in the elderly (20, 64, 65, 67, 72). Their adjuvant use with standard therapy (e.g., nystatin) can improve treatment efficacy and reduce recurrence (72).

Future research should focus on several key directions. Deeper understanding of the interaction network between salivary sIgA and the broader oral microbiota (beyond *Candida*) is crucial for elucidating its role in maintaining microbial balance. Developing rapid clinical tools to detect both salivary sIgA concentration and function (e.g., antigen-binding affinity, inhibitory activity against microbial adhesion) is essential for accurately assessing mucosal immunity and infection risk. Exploring the genetic basis of individual differences in salivary sIgA secretion and function could explain susceptibility variations and inform personalized strategies. Finally, while promising, sIgA-based immunotherapies face practical challenges, including high production costs, stability in the oral environment, and the need for optimized delivery systems, which require thorough investigation.

## 6 Discussion

Salivary sIgA is a central mediator of oral mucosal immunity and a key factor in maintaining ecological balance, occupying a core position in defense against oral candidiasis (4). It controls commensal *C. albicans* carriage by preventing fungal outgrowth, thereby promoting homeostasis (24, 36, 53). Its multifaceted protective mechanisms include immune exclusion (blocking adhesion), neutralization of virulence factors, microbial agglutination for clearance, biofilm disruption, and immune response modulation (8, 60). However, *C. albicans* employs evasion strategies such as protease-mediated sIgA degradation, antigenic variation, and biofilm formation, complicating the host-pathogen interaction (13, 58, 62).

Both the concentration and functional integrity of salivary sIgA critically determine host susceptibility. Reduced sIgA levels due to salivary gland hypofunction, systemic diseases (e.g., HIV/AIDS, diabetes, Sjögren's syndrome), aging, or medical interventions (e.g., radiotherapy, immunosuppressants) significantly increase candidiasis risk (3, 5, 40). Its levels are closely associated with oral candidiasis and can even serve as a supplementary screening marker for diabetes—a major risk factor itself (55). Importantly, functional quality (e.g., antigen-binding avidity) is as clinically significant as quantity; functional impairment can cause recurrent infection despite normal or elevated sIgA levels, underscoring the need to assess both “quantity” and “quality” (56, 57).

Given its pivotal role, enhancing sIgA function offers promising immunotherapeutic avenues. Strategies include passive immunization using topical high-titer anti-*Candida* sIgA (e.g., recombinant sIgA) (41), active mucosal vaccination to induce specific sIgA production (e.g., NDV-3A targeting Als3) (53), and probiotic adjuvants that boost sIgA secretion and restore microbial balance (64, 72). These approaches address limitations of conventional antifungals and open new paths for personalized management. In summary, salivary sIgA is a central mediator of oral mucosal antifungal immunity, and targeting sIgA pathways holds great promise for improving oral candidiasis management, especially in high-risk populations.
